# A subgroup of pancreatic adenocarcinoma is sensitive to the 5-aza-dC DNA methyltransferase inhibitor

**DOI:** 10.18632/oncotarget.2685

**Published:** 2014-12-03

**Authors:** Odile Gayet, Celine Loncle, Pauline Duconseil, Marine Gilabert, Maria Belen Lopez, Vincent Moutardier, Olivier Turrini, Ezequiel Calvo, Jacques Ewald, Marc Giovannini, Mohamed Gasmi, Erwan Bories, Marc Barthet, Mehdi Ouaissi, Anthony Goncalves, Flora Poizat, Jean Luc Raoul, Veronique Secq, Stephane Garcia, Patrice Viens, Nelson Dusetti, Juan Iovanna

**Affiliations:** ^1^ Centre de Recherche en Cancérologie de Marseille (CRCM), INSERM U1068, CNRS UMR 7258, Aix-Marseille Université and Institut Paoli-Calmettes, Parc Scientifique et Technologique de Luminy, Marseille, France; ^2^ Hôpital Nord, Marseille, France; ^3^ Institut Paoli-Calmettes, Marseille, France; ^4^ Centre Génomique du Centre de recherche du CHUL Research Center, Quebec, Canada; ^5^ Hôpital Nord, Département de Gastroentérologie, Marseille, France; ^6^ Hôpital de la Timone, Marseille, France

## Abstract

Pancreatic Ductal Adenocarcinoma (PDAC) is a disease with a great heterogeneity in the response to treatments. To improve the responsiveness to treatments there are two different approaches, the first one consist to develop new and more efficient drugs that intent to cure all patients and the second one is to use already-approved drugs, alone or in combination, but selecting beforehand the most sensitive patients. In this work we explored the efficiency of the second possibility. We developed a collection of 17 PDAC samples collected by Endoscopic Ultrasound-Guided Fine-Needle Aspiration (EUS-FNA) or surgery and preserved as xenografts and as primary cultures. This collection was characterized at molecular level by a transcriptomic analysis using an Affymetrix approach. In this paper we present data demonstrating that a subgroup of PDAC responds to low doses of 5-aza-dC. These tumors show a specific RNA expression profile that could serve as a marker, but there is no correlation with *Dnmt1*, *Dnmt3A* or *Dnmt3B* expression. Responder tumors corresponded to well-differentiated samples and longer survival patients. In conclusion, we present data obtained with the well-known drug 5-aza-dC as a proof of concept that a drug that seems to be inefficient in solid tumors in general could be applicable to a particular subgroup of patients with PDAC.

## INTRODUCTION

Pancreatic adenocarcinoma (PDAC) is the fourth leading cause of cancer death, with a median survival of 6 months and a dismal 5-year survival rate of less than 5%. Importantly, PDAC could move up to the second place as cause of cancer death as early as 2020 according to a new report from the Pancreatic Cancer Action Network. Moreover, using information from the Surveillance Epidemiology and End Results database, in 2030 the number of patients with a PDAC will represent more than a two-fold increase over the current rate in the occidental world [1]. Based on these data the number of deaths from PDAC will exceed those from breast and colorectal cancer, and will be surpassed only by the loss of life from lung cancer.

PDAC is one of the most intrinsically drug-resistant tumors and resistance to chemotherapeutic agents is a major cause of treatment failure. Gemcitabine is the standard chemotherapeutic drug for patients with advanced pancreatic cancer after a phase III trial performed in 1997 that demonstrated a modest survival advantage of this agent over 5-FU. Surprisingly, the most important improvement associated to this treatment was alleviation of disease-related symptoms [2]. More recently, a polychemotherapy regimen combining 5-FU, irinotecan, and oxaliplatin (FOLFIRINOX) was shown to nearly double overall survival compared to gemcitabine, at the expense of a manageable but increased toxicity, limiting its use to good performance status patients. Nevertheless, overall survival was less than 12 months [3]. Therefore, there is a dire need for designing new and targeted therapeutic strategies that can overcome the drug-resistance and improve the clinical outcome for patients diagnosed with pancreatic cancer.

Notably, the great heterogeneity in the response to treatments between patients could be based not only on the differences between hosts but also between PDAC intrinsic characteristics, indicating the existence of many PDAC sub-types. For example, the objective response rate was 31.6% in the multidrug FOLFIRINOX protocol and only 9.4% for patients treated with gemcitabine showing that near to 70% and 90% of patients are not responders respectively [3]. This responsiveness to the treatment is not predictable except for the unresponsiveness to the Gemcitabine treatment in the absence of the specific transporter hENT1 expression [4, 5]. Two different and opposite approaches could be developed to improve the responsiveness to the treatments, the first one consists in developing new and more efficient drugs intending to cure all patients. This approach is extremely expensive and the result uncertain. The second one is based on already-approved drugs, used alone or in combination, selecting beforehand the most sensitive patients. This option presents many advantages including the fact that drugs are easily available, low costs of treatment, toxicity is well known, and the preclinical trials almost unnecessary. In agreement with this second approach, we developed a collection of 17 PDAC samples from 17 consecutive patients of whom we have the complete clinical outcome. The tumors were preserved as xenografts and as primary cultures allowing the growth of cancerous epithelial cells only. This collection, which was well characterized at the molecular level, serves us to test the tumor sensitivity to different known drugs and as an important source of molecular markers.

DNA methylation is an epigenetic modification that maintains DNA transcriptionally quiescent causing gene silencing. DNA methyltransferases (DNMTs) are the enzymes that catalyze the addition of methyl groups to the 5′ carbon of the cytosine residues [6]. Several isoforms of DNMTs are present in cells. DNMT1 is associated to the maintenance of established patterns of methylated DNA, while DNMT3A and DNMT3B seem to mediate de novo DNA methylation patterns [7, 8]. Many different DNMT inhibitors have been developed (nucleosides analogues such as Azacitidine, Decitabine and Zebularine or non-nucleosides analogues such as MG98, RG108 and Procainamide) and multiple molecular mechanisms by which DNMT inhibitors induce anti-cancer effects have been identified, in most cases by the modulation of specific genes involved in cellular processes such as apoptosis, cytostasis, differentiation and tumor angiogenesis. Therefore, it is not surprising that DNMT inhibitors are emerging as promising class of drugs in cancer treatment, especially in combination with other agents or with other treatments.

5-aza-dC is a DNMT inhibitor incorporated into DNA as a deoxycytidine analog that forms irreversible covalent bonds with DNMT at cytosine sites targeted for methylation [9]. 5-aza-dC demonstrates activity against hematologic malignancy [10] and is used as first-line of treatment in AML patients > 65 years who are not candidates for intensive chemotherapy [11], whereas its efficacy in solid tumors is very limited [12]. Clinical responses appear to be exerted both by epigenetic alterations and by induction of cell-cycle arrest and/or apoptosis [13, 14]. In this paper we present data demonstrating that a subgroup of PDAC responds to low doses of 5-aza-dC, these tumors show a specific RNA expression profile that could serve as a marker, but there is no correlation with *Dnmt1*, *Dnmt3A* or *Dnmt3B* expression. Responder tumors corresponded to well-differentiated samples and longer survival patients. In conclusion, we present the data obtained with the well-known drug 5-aza-dC as a proof of concept that a drug that seems to be inefficient in solid tumors in general could be applicable to a particular subgroup of patients.

## RESULTS

### Chemogram to the 5-aza-dC compound

**Figure 1:**
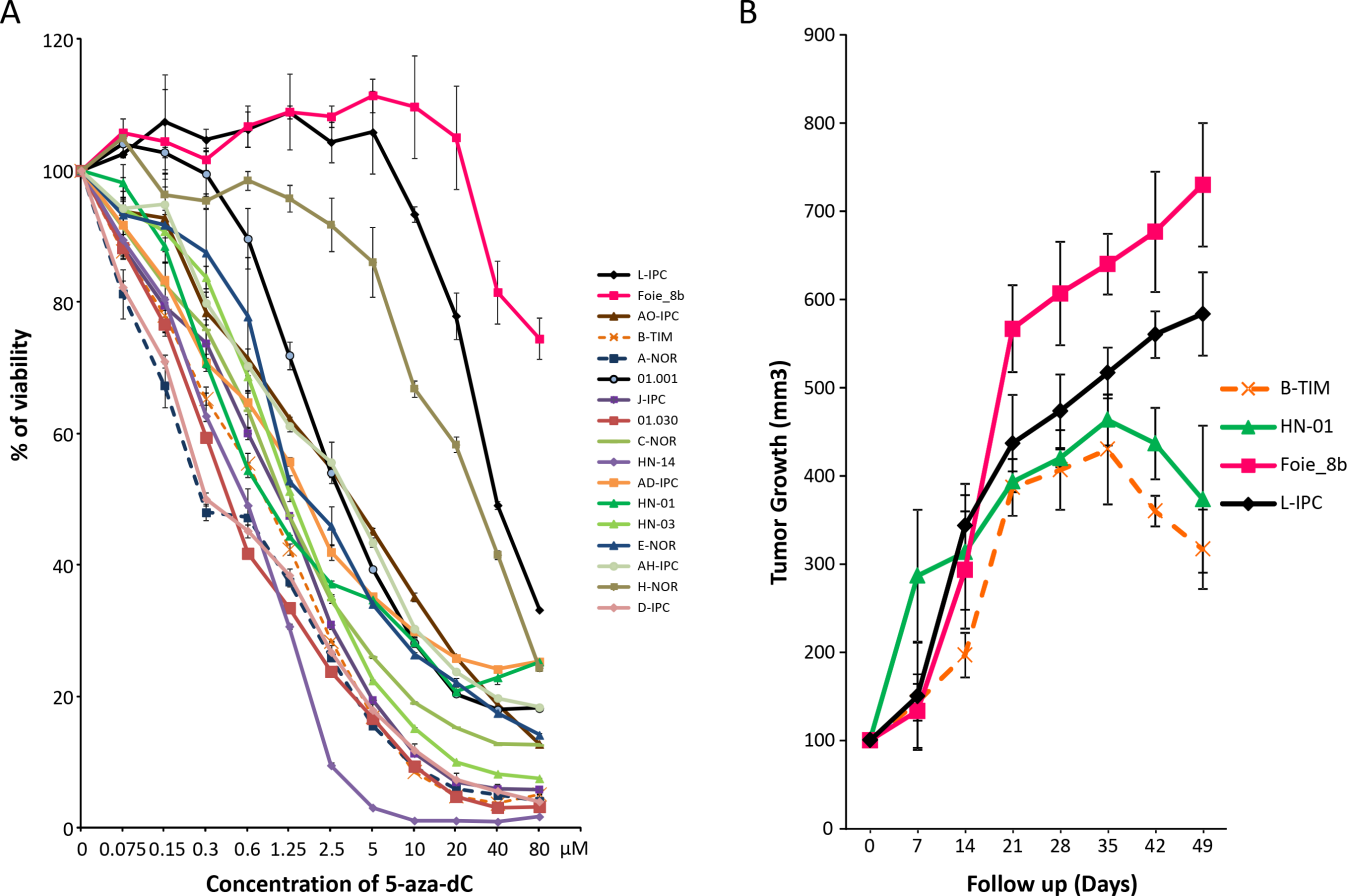
Sensitivity to the 5-aza-dC *in vitro* and *in vivo* **(A)** Chemogram of 5-aza-dC. PDAC-derived cells were treated with increasing concentrations of 5-aza-dC and the survival cells were measured after 72 h of treatment. **(B)** *in vivo* 5-aza-dC sensitivity. PDAC xenografted mice were treated with 5-aza-dC and tumor measured weekly. Treatment starts at day 21 after implantation. In grey is marked the length of the treatment. Error bars ± SEM; *n* = 3 per group.

Primary cell cultures from patient's pancreatic tumors were submitted to increasing concentrations (from 0 to 80 μM) of 5-aza-dC in order to study their sensitivity and to obtain a dose-response curve. Using this approach we were able to compare 17 different PDAC-derived primary cultures estimating their relative chemosensitivity. As shown in Figure 1A and Table 1, each patient-derived primary culture shows a different pattern of chemosensitivity. Whereas A-NOR (DL50 = 0.29 μM), D-IPC (DL50 = 0.29 μM) and 01.030 (DL50 = 0.50 μM) are the 3 most sensitive patient derived primary cultured cells to 5-aza-dC; the Foie_8b (DL50 >80 μM), L-IPC (DL50 = 41.0 μM) and H-NOR (DL50 = 33.0 μM) are the most resistant to the 5-aza-dC compound. The sensitivity of two resistant primary cultures (L-IPC and Foie-8b) and two sensitive (HN-01 and B-TIM) were tested *in vivo* and results showed in Figure 1B. To this end, four tumors (100 mm^3^) corresponding to two sensitive (HN-01 and B-TIM) and two resistant (L-IPC and Foie_8b) primary cultures were xenografted in nude mice. Treatment with 5-aza-dC was started at day 21 after implantation and for a period of 4 weeks. Tumor growth curves confirm that L-IPC and Foie-8b xenografts are 5-aza-dC resistant tumors whereas HN-01 and B-TIM xenografts are more sensitive in agreement with data obtained *in vitro*.

**Table 1:** DL50 values corresponding to 17 pancreatic cancer-derived cells

Cell	DL50
01.001	3.10
01.030	0.50
AD-IPC	1.80
AH-IPC	3.80
A-NOR	0.29
AO-IPC	3.90
B-Tim	0.90
C-NOR	1.26
D-IPC	0.29
E-NOR	1.83
Foie_8b	>80
H-N01	0.91
H-N03	1.30
H-N14	0.63
H-NOR	33.0
J-IPC	0.58
L-IPC	41.0

Altogether, these results show that PDAC-derived primary cultures present variable sensitivities to 5-aza-dC with DL50s ranging from 0.29 to > 80 μM which is a range of more than 320 folds and that *in vitro* experiments correlates with the *in vivo* treatments. This strong variability encourages us to go forward with this study trying to find molecular markers that may identify sensitive patients.

### Correlation between transcriptome and 5-aza-dC sensitivity in PDAC

**Figure 2:**
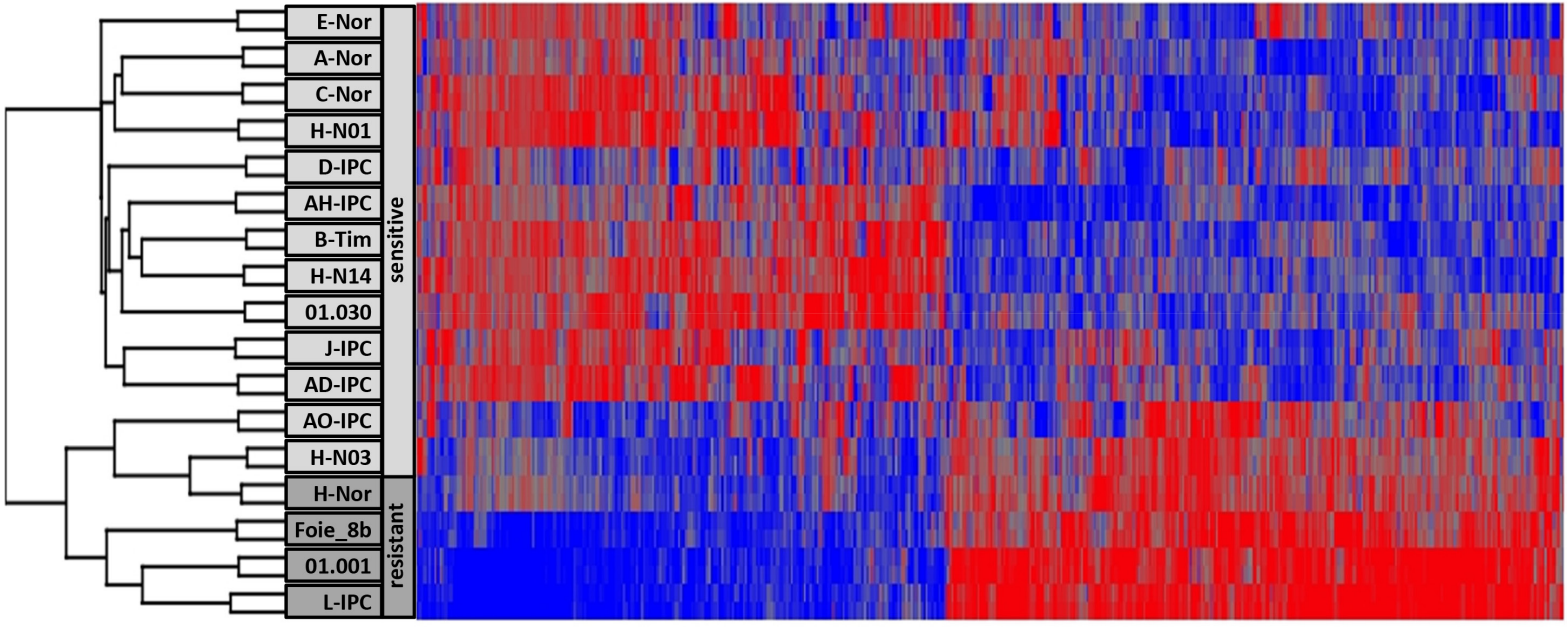
RNA expression analysis of PDAC The Heatmap showing the RNA expression profile of PDAC sensitive and resistant to the 5-aza-dC treatment.

To study whether or not there is a correlation between 5-aza-dC response and transcriptome, we performed a heatmap analysis on the transcriptome of xenografted human PDAC by clustering tumors according to their expression profiles. To our surprise, the 5 more resistant PDAC (Foie_8b, L-IPC, H-NOR, AO-IPC and 01.001) and the moderately resistant HN-03 (DL = 1.30 μM) appear in a relatively homogeneous group of tumors whereas the other 12 more sensitive PDAC form a relatively distant group of tumors as shown in Figure 2 and Supplementary Tables 1 and 2.

Among the transcripts identified in this phenotype, 326 corresponded to upregulated whereas 381 to downregulated genes involved in several pathways. Among these data, we observed that resistant tumors are undifferentiated or poorly differentiated contrary to the sensitive tumors that are well or middle differentiated. We observed that TGFB1 and its target gene TGFBI are overexpressed in resistant tumors by 3.24 and 3.08 fold. Their strong involvement in epithelia to mesenchymal transition process may explain the accrue resistance to the treatment.

Altogether, these results suggest that a specific tumor phenotype could be associated with resistance or sensitivity to an anticancer drug which could be of clinical interest.

### Sensitivity to 5-aza-dC in PDAC does not correlate with expression of *Dnmt1*, *Dnmt3A* or *Dnmt3B*

**Figure 3:**
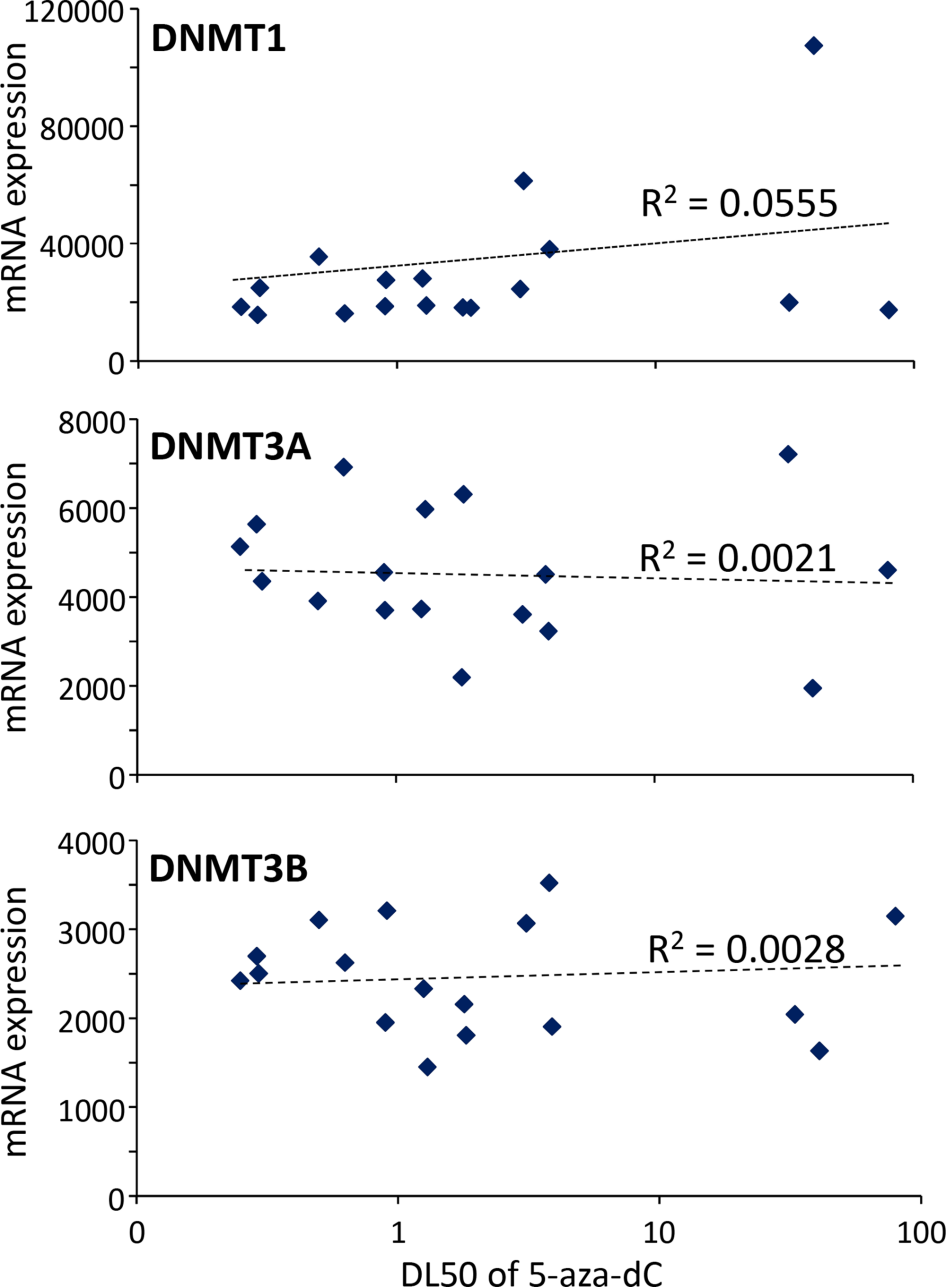
Correlation between sensitivity of the 5-aza-dC and expression of DNMT1, DNMT3A and DNMT3B transcripts DL50 of 5-aza-dC was calculated for each xenograft-derived cell and correlated with expression of DNMT1, DNMT3A and DNMT3B mRNAs. r^2^ is the correlation coefficient.

Because, on one hand, the 5-aza-dC is an inhibitor of the DNMT1 activity and, on the other hand, it was proposed a relationship between sensitivity to the drug and DNMT1 expression in PDAC cells, we analyzed the correlation between DNMT1 as well as DNMT3A- and DNMT3B-related enzymes with the sensitivity of the cells to the treatment with 5-aza-dC. To this end we measured the expression of all this three transcripts in the RNA purified from the xenografts and we correlated these data with the DL50 obtained on the xenograft derived primary cultures. As shown in Figure 3, the R^2^ for DNMT1 is 0.0555, for DNMT3A is 0.0021 and for DNMT3B is 0.0028 which are extremely low indicating no correlation. These results strongly suggest that the sensitivity to 5-aza-dC is independent of the DNMTs expression levels and therefore measuring their levels is clinically irrelevant.

### Correlation between sensitivity to the 5-aza-dC and the clinical outcome

**Figure 4:**
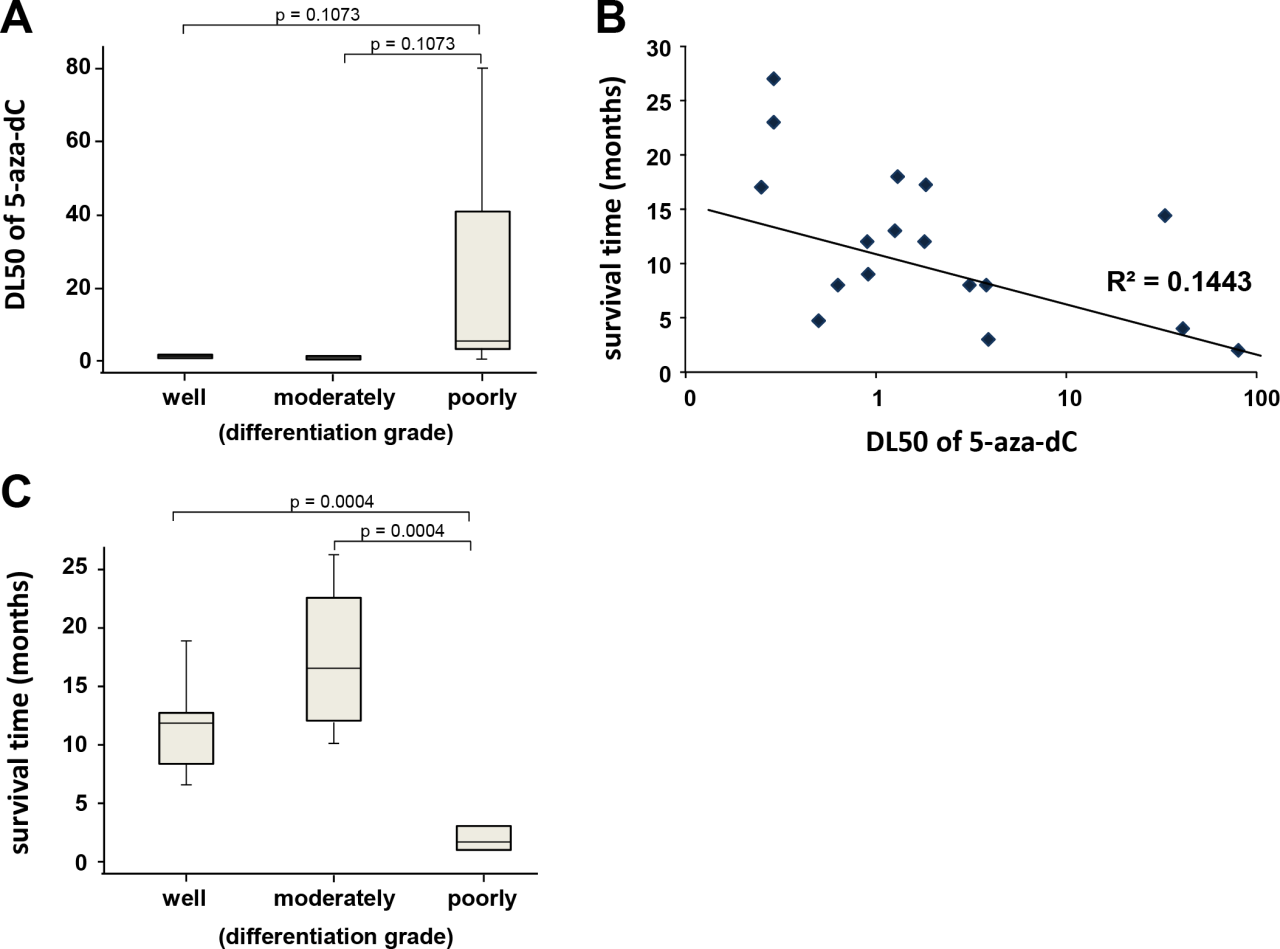
(A) Box and whiskers plot showing the distribution of DL50 for 5-aza-dC in a three point score of tumor differentiation (well, moderately and poorly differentiated or undifferentiated) **(B)** Correlation between sensitivity of the 5-aza-dC and survival time of patients. DL50 of 5-aza-dC was calculated for each xenograft-derived cell and correlated with the time of survival for each patient in months. r^2^ is the coefficient of correlation. **(C)** Box and whiskers plot showing the distribution of patient's survival time in a three point score of tumor differentiation.

From a histopathological point of view, we observed that resistant tumors are undifferentiated or poorly differentiated and that sensitive tumors are well or moderately differentiated (Figure 4A). The sensitivity curve to the 5-aza-dC treatment was obtained from 17 primary cultured cells derived from the xenografts and a putative correlation with the clinical outcome of patients was analyzed. Data presented in Figure 4B indicate a modest correlation with the survival time of patients. These results are in agreement with the fact that patients with undifferentiated and poorly differentiated tumors have a shorter survival time as presented in Figure 4C. These observations suggest that patients with a well differentiated PDAC should be more sensitive to the treatment with 5-aza-dC and probably with other DNMTs inhibitors. Altogether, our results suggest that because of the heterogeneity of PDAC, a subgroup of patients with well differentiated tumors, are more sensitive to 5-aza-dC than undifferentiated tumors. An additional work including a larger number of patients will be necessary to confirm this observation, however, these results validate the concept according to which studying the sensitivity of PDAC derived cells to a set of drugs could allow to define a line of treatment more adapted to the patient.

## DISCUSSION

The main goal of this study was to define whether there are differences in PDAC sensitivity to drugs and in such case, if there is a possibility to identify the most sensitive patients by using specific markers that arise from the molecular characterization of their tumors. The results presented in this paper suggest that there is a group of tumors sensitive to the well-known drug 5-aza-dC. 5-aza-dC is an interesting compound since its mechanism of action is well known and several clinical trials have been performed in patients with different tumors, including several solid tumors.

The rational to use methyltransferases inhibitors to treat tumors is that neoplastic cells exhibit global hypomethylation with localized hypermethylation of CpG islands and increased levels of methyltransferases activity [15]. Moreover, aberrant hypermethylation of CpG islands is associated with transcriptional silencing of genes, which not only plays a role in tumorigenesis, but may also influence response to anticancer agents [16, 17]. Therefore, reversing gene methylation and epigenetic silencing has the potential to influence tumor growth, sensitivity to anticancer agents, and ultimately clinical outcome [18]. Several studies have documented the relevance of epigenetic alterations in pancreatic cancer and the effect of 5-aza-dC on cell coming from this tumor [19–21]. In clinical trials, although the 5-aza-dC has shown an objective response in some patients, its overall efficiency remains relatively low. For these reason 5-aza-dC is not used in the treatment of pancreatic cancers. This is a proof of concept study that intent to demonstrate that drugs that present an interest in a particular sub-group of patients should be studied by identifying the sensitive tumors with specific markers.

Surprisingly, there is no correlation between sensitivity to 5-aza-dC and DNMT1, neither with DNMT3A or DNMT3B, expression level but a significant correlation was observed with an expression profile specifically associated to sensitivity. In addition, expression of other DNA methylation associated molecules such as Mecp2 (methyl CpG binding protein 2) or Polycomb-group proteins including SUZ12, Eed, Ezh1 and Ezh2 do not correlate with the 5-aza-dC sensitivity (data not shown). These results are interesting and original because they show that effect of the drug is not dependent of its target level and they indicate that sensitivity is dependent on other cellular mechanisms. This is not surprising since there are no reports in the literature associating efficiency of 5-aza-dC and levels of DNMT1 expression in tumors except data obtained by Li and colleagues which conclude that PDAC-derived cells with low DNMT1 expression tend to be more sensitive to low dose of 5-aza-dC [22]. Altogether, these results strongly suggest that there is no correlation or if any it is only small.

One of the most interesting points from this work is the fact that we used an efficient strategy in which PDAC tumors from 17 consecutive patients were collected from surgical specimens and EUS-FNA biopsies by xenografting in immunosuppressed mice. We studied the RNA expression profile by a microarray approach on these xenografts, rather than in the cultured cells, since morphology and microenvironment, both playing determinant effect on RNA expression, were closer to the original human PDAC as recently confirmed [23]. Then, primary culture of cells obtained from xenografts allowed us to analyze their relative sensitivity to 5-aza-dC *in vitro*. All these data were used to detect a possible correlation between drug responsiveness and RNA expression profile. Surprisingly, we identified a profile of genes which significantly correlates with the sensitivity to the treatment suggesting that the profile of expression can be used to identify tumors types and thereby establish groups of tumors with more or less sensitivity to the drug. We are aware that the number of genes we found defining sensitivity to 5-aza-dC treatment is quite high but after increasing the number of PDAC samples will probably allow us to detect the most efficient and robust genes. Therefore, we can expect that increasing the number of patients will increase the sensitivity and specificity with a more limited number of genes to constitute the signature determining drug sensitivity and consequently patient outcome.

Another interesting point to be noted is that sensitivity to 5-aza-dC treatment correlates with the long-term survival patients carrying well- and moderately-differentiated tumors. This is in agreement with the fact that some genes typically expressed in poorly-differentiated PDAC such as MUC3A (13.3 fold increase), MUC5AC (9.76 fold increase), GATA6 (5.57 fold increase), or HNF4A (4.43 fold increase) are differentially overexpressed in sensitive compared to resistant PDAC-derived cells (Supplementary Table 2). This data strongly suggest that 5-aza-dC treatment is more efficient against well- and moderately-differentiated tumors than against the poorly-differentiated ones. Interestingly several studies have shown that after resection of pancreatic cancer, a measure of the tumor differentiation degree is an important prognostic indicator, in general, the more undifferentiated the tumor, the more aggressive the malignant biology [24].

In conclusion, we showed in this work that expression profiling of xenografts from PDAC patients is able to discriminate sensitive from resistant tumors. This fact should be used to aid to take decision to treat patients with a PDAC.

## METHODS

### PDAC samples and cell culture

Consent's forms of informed patients were collected and registered in a central database. The tumor tissues used for xenograft development was deemed excess to that required for the patient's diagnosis. Two types of samples were obtained, namely Endoscopic Ultrasound-Guided Fine-Needle Aspiration (EUS-FNA) biopsies from patients with unresecable tumors, and tumor tissues from patients undergoing surgery. PDAC samples were mixed with 100 μl of Matrigel (BD Biosciences) and implanted with a trocar (10 Gauge, Innovative Research of America, Sarasota, FL) in the subcutaneous right upper flank of an anesthetized and disinfected mouse. When tumors reached 1 cm^3^, mice were sacrificed and tumors were removed.

To obtain primary cell cultures of these tumors, xenografts were splited into several small pieces and processed in a biosafety chamber: after a fine mincing, they were treated with collagenase type V (ref C9263; Sigma) and trypsin/EDTA (ref 25200-056; Gibco, Life Technologies) and suspended in DMEM supplemented with 1% w/w Penicillin/Streptomycin (Gibco, Life Technologies) and 10% Fetal Bovine Serum (Lonza). After centrifugation, cells were re-suspended in Serum Free Ductal Media (SFDM) adapted from Schreiber et al. [25] and incubated at 37°C in a 5% CO_2_ incubator.

### Gene expression microarrays

RNAs extraction was performed according to Chirgwin's protocol (Chirgwin et al., 1979). Total RNA (1.0 μg) was reverse transcribed for hybridization to the human oligonucleotide array Human Gene 2.0 (Genechip, Affymetrix) as described previously [26]. Briefly, arrays were processed using the Affymetrix GeneChip Fluidic Station 450 (protocol EukGE-WS2v5_450) and scanned using a GeneChip Scanner 3000 G7 (Affymetrix). The GeneChip Operating Software (Affymetrix GCOS v1.4) was used to obtain chip images and for quality control. Background substraction and normalization of probe set intensities were performed using the method of Robust Multiarray Analysis (RMA) [27]. All array data are available at National Center for Biological Information (NCBI) Gene Expression Omnibus (GEO) omnibus GSE55513.

### Chemogram

Cells were screened for their chemosensitivity to 5-aza-dC (ref. A2385 Sigma Aldrich). These cells were treated for 72 h with increasing concentrations of 5-aza-dC ranging from 0 to 80 μM. Five thousand cells per well were plated in 96-wells plates in SFDM medium. Twenty four hours later the media was supplemented with increasing concentrations of 5-aza-dC and incubated for an additional 72 h period. Each experiment was done in triplicate and repeated at least three times. Cell viability was estimated after addition of PrestoBlue reagent (Life Technologies) for 3 h following the PrestoBlue cell viability reagent protocol provided by the supplier.

### RT-qPCR

One μg RNA from xenografts was reversed transcribed using the Go Script reagent (Promega) according to manufacturer's instructions. Real-time quantitative PCR for DNMT1, DNMT3A and DNMT3B mRNA was performed in a Stratagene cycler using Takara reagents. Primers sequences are available upon request.

### *In vivo* experiments

*In vivo* experiments were conducted in accordance with institutional guidelines and were approved by the “Plateforme of Stabulation et d'Expérimentation Animale” (PSEA), Scientific Park of Luminy, Marseille. Human-PDAC xenografts were established by subcutaneous implantation of human tumors in the upper right flank of 5- to 6-week-old nude mice nude mice (Swiss Nude Mouse Crl: NU(lco)-Foxn1nu, Charles River Laboratories). Matrigel (BD Biosciences) was added to tumors just before implantation and tumors were maintained in mouse by splitting. For the 5-aza-dC (Sigma) treatment the drug was injected at 0.250 mg/kg/daily intraperitoneally (150 μl per injection) starting at day 21 after implantation. A fragment of 100 mm^3^ was implanted and the growing of tumors was measured weekly by caliper and calculated as length x width x depth.

### Clinical outcome

The 17 patient's clinical outcomes were recorded in a prospective database, and data such as age, gender, medical history, histopathological stage of differentiation, treatments received, response to chemotherapy, date of progression, metastasis occurrence and localization and death date were analyzed. Progression-free survival and overall survival were then calculated.

### Differentiation score

PDAC xenograft score of differentiation was based on the extent of glandular differentiation. If  > 95% of the tumor is composed of glands then it is classified as being well differentiated, 50%–95% is moderately differentiated, and < 50% is poorly differentiated or undifferentiated.

## SUPPLEMENTARY TABLES






