# Supplementary material for: A subgroup of pancreatic adenocarcinoma is sensitive to the 5-aza-dC DNA methyltransferase inhibitor
Source: Oncotarget. 2014 Dec 3;6(2):746–54. doi: 10.18632/oncotarget.2685 (PMC4359252; doi:10.18632/oncotarget.2685)
Supplement: Supplementary file 1 [file oncotarget-06-746-s001.pdf]

SUPPLEMENTARY TABLES

Supplementary Table S1. Genes underexpressed in sensitive compared to resistant tumors to 5-aza-dC

Supplementary Table S2. Genes overexpressed in sensitive compared to resistant tumors to 5-aza-dC
